# Supplementary figures and images for: Estimating pulse wave velocity from the radial pressure wave using machine learning algorithms
Source: PLoS One. 2021 Jun 28;16(6):e0245026. doi: 10.1371/journal.pone.0245026 (PMC8238176; doi:10.1371/journal.pone.0245026)

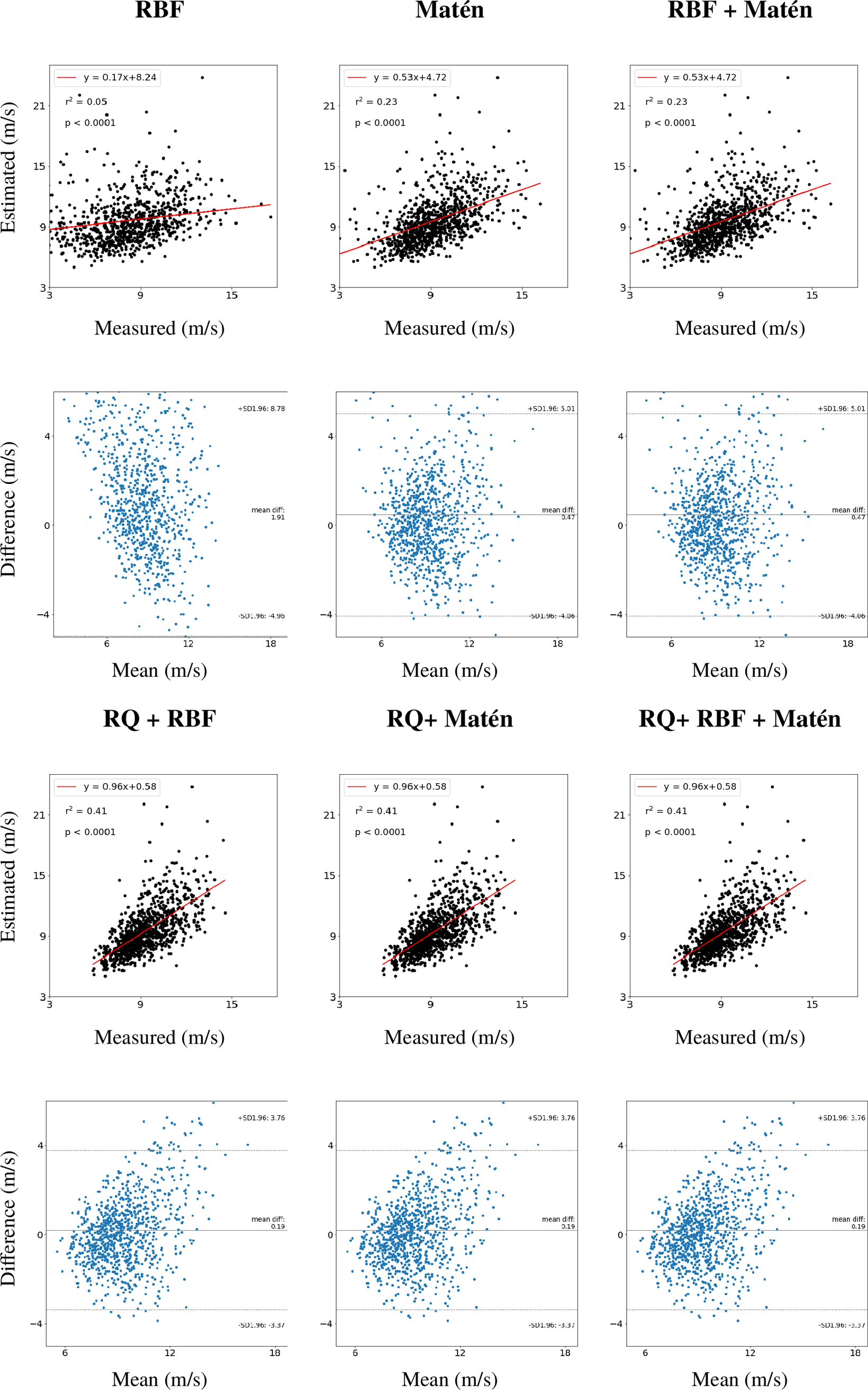

Supplement: S1 Fig — RBF: radial basis function; Matérn: Matérn kernel; RQ: rational quadratic kernel. (TIF) [file pone.0245026.s001.tif]

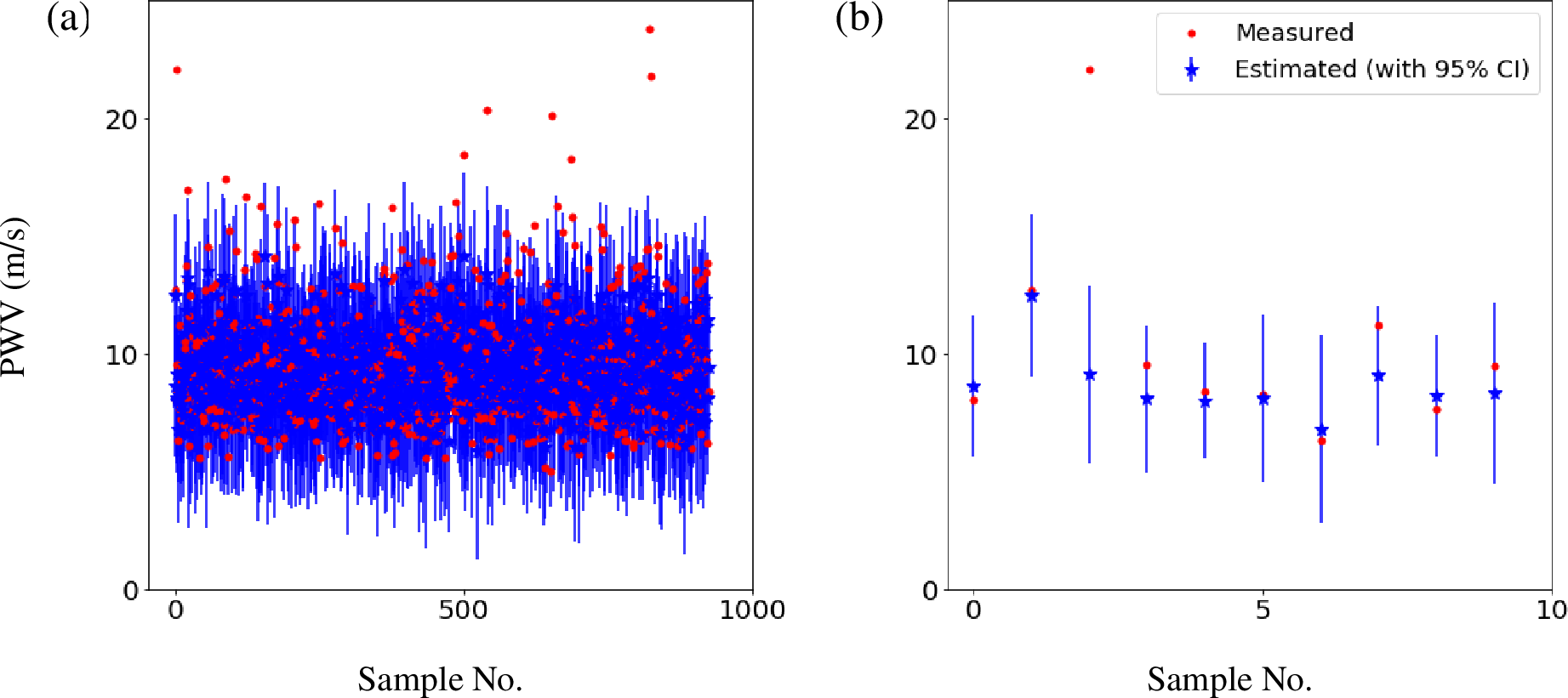

Supplement: S2 Fig — Panel (a) shows the measured and estimated PWV plot on top of each other; panel (b) shows the first ten samples in panel (a). (TIF) [file pone.0245026.s002.tif]

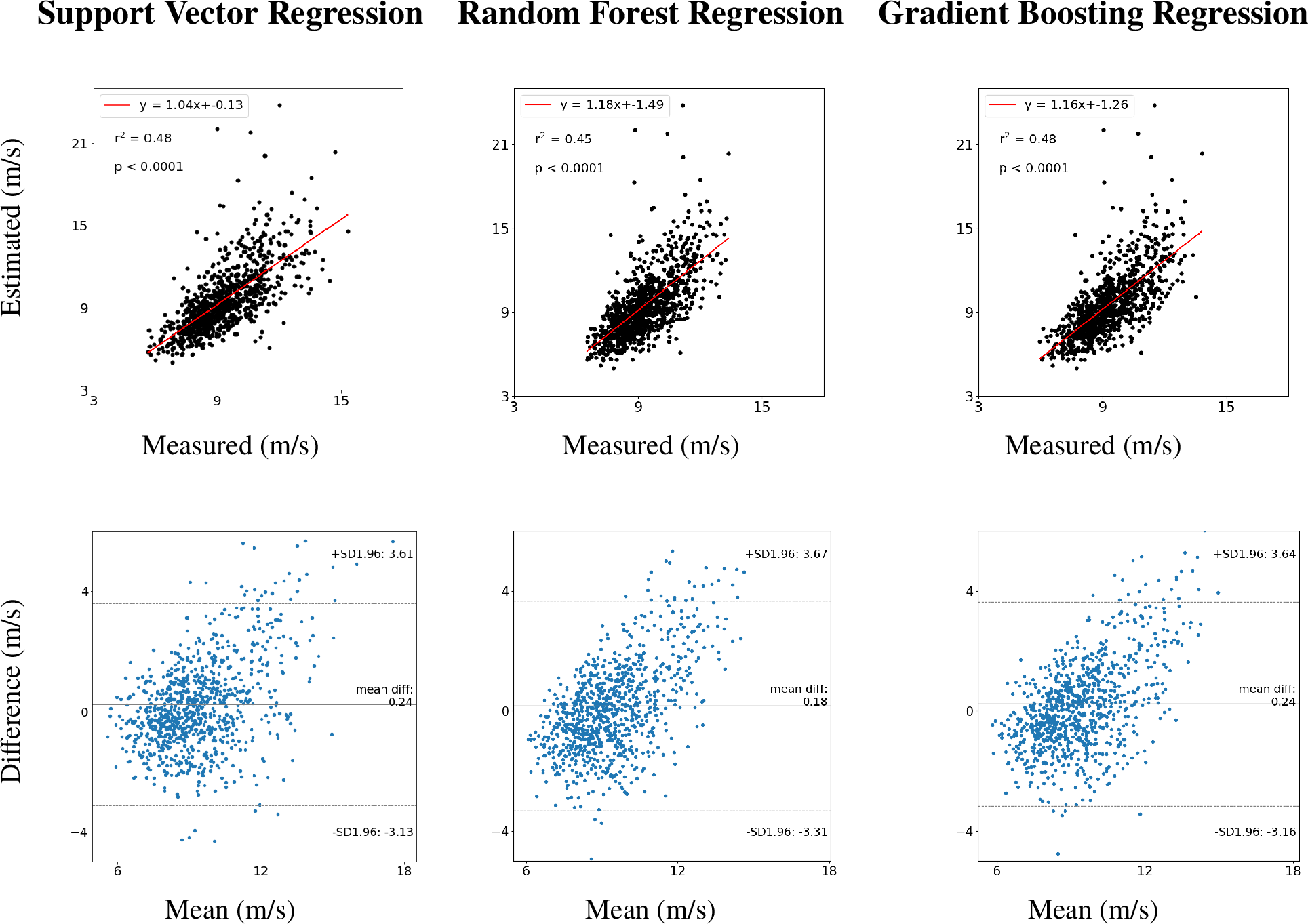

Supplement: S3 Fig — (TIF) [file pone.0245026.s003.tif]

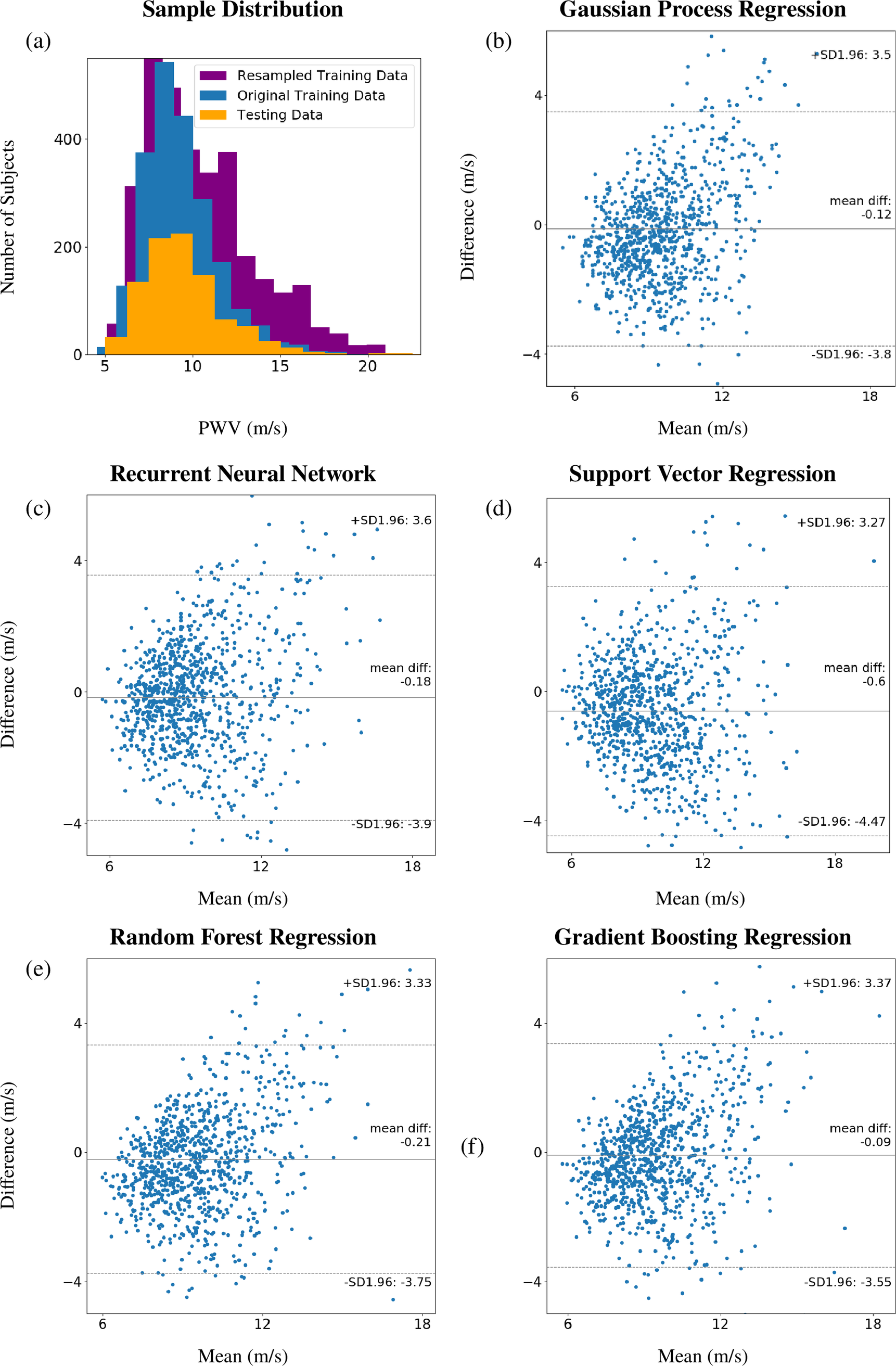

Supplement: S4 Fig — (TIF) [file pone.0245026.s004.tif]

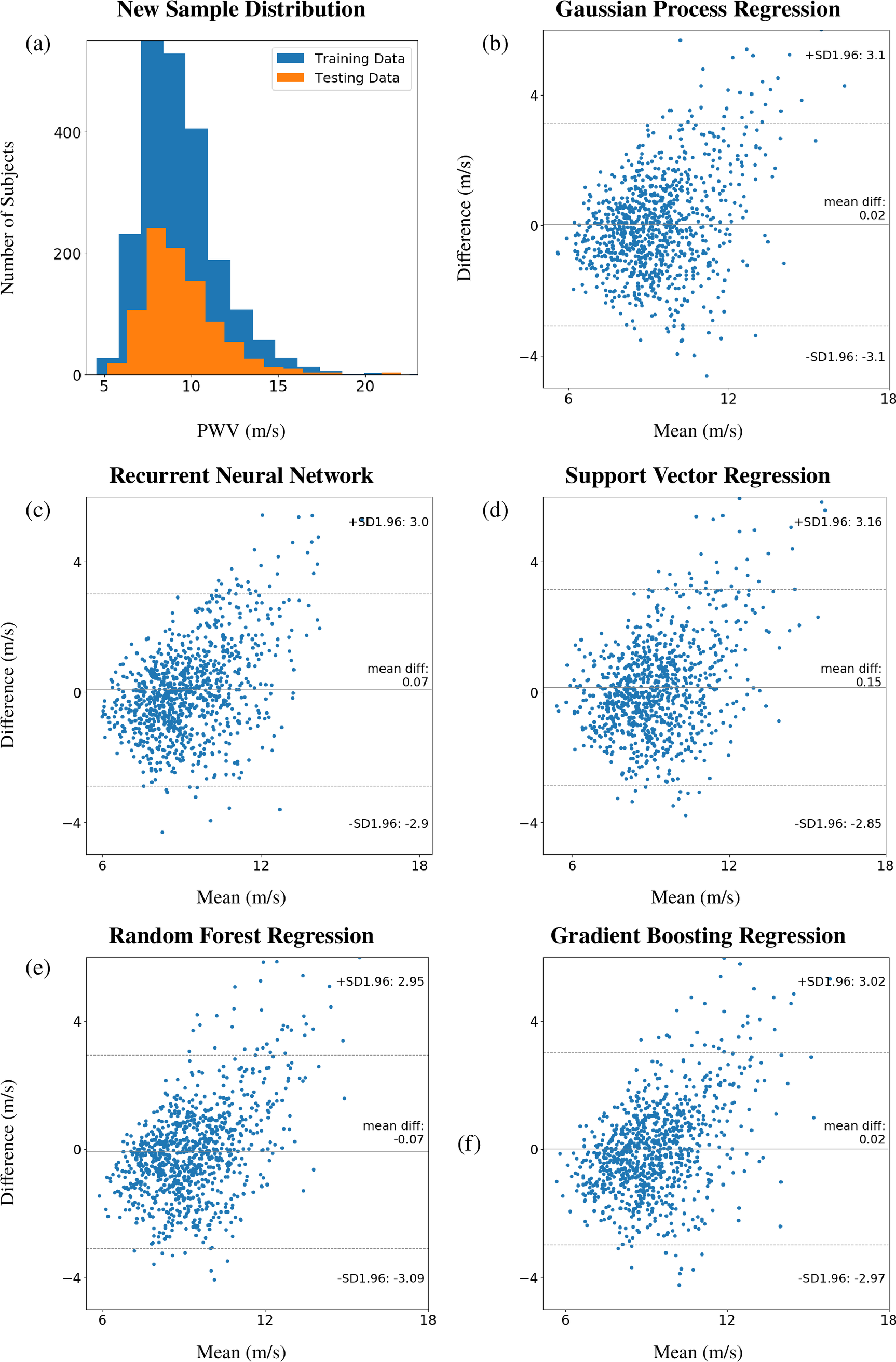

Supplement: S5 Fig — (TIF) [file pone.0245026.s005.tif]
